# Influence of Different Sweeteners on the Stability of Anthocyanins from Cornelian Cherry Juice

**DOI:** 10.3390/foods9091266

**Published:** 2020-09-10

**Authors:** Bianca Moldovan, Luminita David

**Affiliations:** Research Center for Advanced Chemical Analysis, Instrumentation and Chemometrics (ANALYTICA), Faculty of Chemistry and Chemical Engineering, “Babeş-Bolyai” University, 11 Arany Janos Street, 400028 Cluj-Napoca, Romania; bianca@chem.ubbcluj.ro

**Keywords:** Cornelian cherries, anthocyanins, fructose, sucrose, aspartame, acesulfame potassium

## Abstract

Cornelian cherries are red fruits which can be considered as a valuable dietary source of antioxidant biologically active compounds, especially anthocyanins. The purpose of the present study was to investigate the anthocyanins degradation process in Cornelian cherry juice supplemented with different sweeteners. Four formulations of Cornelian cherry juice were prepared using different sugars (sucrose, fructose) or artificial sweeteners (aspartame and acesulfame potassium). The obtained juices were stored at three distinct temperatures (2 °C, 25 °C, and 75 °C) in order to evaluate the effects of the sweetener and storage conditions on the pigment stability. The rate constants (k) and the half time values (t_1/2_) of the degradation processes were determined. The highest stability was observed for the anthocyanins from the unsweetened juice stored at 2 °C (k = 0.5·10^−3^ h^−1^), while the most accelerated degradation was registered for the fructose sweetened juice stored at 75 °C (k = 91.65·10^−3^ h^−1^). The presence of the different sweeteners in the Cornelian cherry juice affects their pigment stability during storage. The highest change in the retention of anthocyanins was determined by the presence of fructose, while acesulfame potassium had the less deleterious effect.

## 1. Introduction

Cornelian cherries are red fruits which can be considered as a good source of health-promoting phytocompounds, such as phenolics, flavonoids, iridoids, and vitamin C, which contribute to their nutritive value and organoleptic properties and strongly influence their quality and consumers acceptance [1,2,3]. The attractive red color of Cornelian cherries results from their high amount of anthocyanic pigments, which also confer numerous health benefits to these fruits, such as controlling obesity, preventing cardiovascular disorders, diminishing diabetes deleterious effects, reducing inflammation by inhibiting COX enzymes, presenting anticarcinogenic properties, and reducing the viability of severe human pathogens [4,5]. As a consequence of all these properties, there is an increasing demand for both red fresh fruits and products derived from them, as consumers demand foods that positively affect their health. Cornelian cherry fruits can be processed by several methods into jams, jellies, marmalades, yogurts, or beverages and juices [6,7,8].

Processing and storage conditions may strongly affect the juice quality parameters, such as color, antioxidant properties, phenolic, and anthocyanins content. Changes in all these parameters are mainly caused by the change in anthocyanins retention. The stability of anthocyanins can be influenced by many factors, such as heating, pH, oxygen, light, the presence of ascorbic acid, and metal ions. The processing and storage of anthocyanins containing food products can result in pigment degradation, leading to colorless or undesirable brown compounds [9].

The determined main anthocyanins in *Cornus mas* fruits are cyanidin, delphinidin, and pelargonidin glucosides, as follows: cyanidin-3-O-glucoside, cyanidin-3-O-galactoside, delphinidin-3-O-galactoside, pelargonidin-3-O-glucoside, and pelargonidin-3-O-rutinoside [10]. Cornelian cherries’ anthocyanins are known to possess a relatively high stability. The investigation of their degradation in various conditions has shown that this process occurs slower than the loss of anthocyanins from other sources (blackberry, mulberry, etc.) [11,12]. The high stability of the anthocyanin pigments from Cornelian cherry fruits and their high content of bioactive compounds, particularly phenolics, as well as other nutrients, enables us to recommend these fruits for developing healthy foods and beverages.

Health professionals and the new dietary guidelines encourage the industry to find nonsugar alternatives to sweetened foods. Researchers currently focus their activity on developing foods with lower sugar content or with alternative sources of sweeteners due to the rising concern of sugar dietary intake, intake associated with numerous diseases such as obesity, cardiovascular disorders, high cholesterol levels, and diabetes mellitus [13]. The use of noncaloric sweeteners represents an alternative for the beverage industry, but this may affect the sensory characteristics of fruit juices. The sweeteners (sugars or noncaloric sweeteners) may also affect the stability of the health-promoting anthocyanins, so the type of sweetener added to red fruit juices should be carefully selected in order to minimize the degradation rate and color deterioration. Wrolstad et al. [14] demonstrated that sucrose increased strawberry anthocyanins stability, while Kopjar and Pilizota [15] reported that blackberry anthocyanins faster degraded after sucrose addition. Using fructose as a sweetener negatively affected the stability of black currant anthocyanins, while aspartame and sucrose enhanced their stability [16]. All of these previous studies clearly indicate that structure and concentration of the sweetener, as well as temperature and anthocyanins’ nature and composition, affect the stability of pigments from various sources. Thus, investigating sweetener-related anthocyanins thermostability is very important in taking preventive measurements to protect anthocyanins to degrade.

The present study aimed to evaluate the thermal stability of anthocyanins from Cornelian cherry juice at three different storage temperatures: 2 °C (refrigerated storage), 25 °C (room temperature storage), and 75 °C (juice pasteurization temperature) in the presence of two caloric (glucose and fructose) and two noncaloric (aspartame and acesulfame potassium) sweeteners. As data on this specific subject are lacking, this is the first study on the impact of sweeteners on the degradation of anthocyanins in Cornelian cherry juice.

## 2. Materials and Methods

### 2.1. Chemicals

The standards of the anthocyanin compounds (pelargonidin-3-O-rutinoside, pelargonidin-3-O-glucoside and cyanidin-3-O-galactoside) were obtained from Extrasynthese (Lyon, France). Merck (Darmstadt, Germany) provided all other reagents and solvents of analytical or HPLC purity. A TYPDP1500 Water distiller (Technosklo Ltd., Drzkov, Czech Republic) was used to obtain distilled water.

### 2.2. Preparation and Characterization of Fruitjuice

Cornelian cherry (*Cornus mas* L.) fruits were obtained from a local market in Cluj-Napoca, Romania, in August 2019. The fully ripen fruits, according to their dark red color, were selected. Foreign materials were removed, and the selected Cornelian cherries were washed first with cold tap water and then with distilled water. After removal of stones, the fruit pulp was blended with deionized water (1:1 *w*/*w*). The mixture was depectinized with a pectolytic enzyme, Pectinex Ultra SPL (1% *v*/*w*), at 50 °C for 2 h. After that, it was transferred in a laboratory press to obtain fruit juice. The juice was frozen and further analyzed and used for the investigation of anthocyanins stability in various conditions.

The physicochemical parameters of the fruit juice were evaluated. The total content of soluble solids (TSS) was measured using a Bellingham + Stanley refractometer (Bellingham + Stanley Ltd., Kent, UK) at 20 °C and expressed as degrees Brix. An inoLab pHmeter (Veilheim, Germany) was used to measure the pH value.

The anthocyanic pigments from *Cornus mas* fruit juice were identified and quantified by HPLC DAD analysis and pH differential method, respectively [10,17]. The anthocyanins were identified using an Agilent 1200 (Agilent Technologies Inc., Santa Clara, CA, USA) equipped with a diode-array detector chromatograph. The analysis was carried out on an Eclipse XTB-C18 column (inner diameter of 150 × 4.6 mm, 5-µm particle size) at 20 °C. Formic acid aqueous solution (0.1%) was used for the dilution of the juice samples and after that the diluted samples were filtered through a 0.2-µm PTFE membrane disk filter. Samples of 20 µL were injected in the chromatographic system, and the separation was performed using a mixture of 0.1% formic acid aqueous solution and 0.1% formic acid acetonitrile solution and a linear gradient (time, %A): 0, 95%; 16, 60%; 17, 5%; 20, 95%. The flow rate was 0.4 mL/minute, and the detection was performed by recording chromatograms at 506 nm.

The total monomeric anthocyanin content of Cornelian cherry juice was evaluated using two buffer systems: KCl buffer (0.025 M, pH = 1) and CH_3_COONa buffer (0.4 M, pH = 4.5). The juice samples were three-fold diluted with distilled water, and 0.8 mL of diluted samples were mixed with 3.2 mL of each buffer solution. After 15 min of equilibration in the dark at ambient temperature, the absorbance of each sample was determined at λ_max_ = 506 nm and, for haze correction, at 700 nm with a Perkin Elmer Lambda 25 UV-Vis spectrophotometer (Perkin Elmer, Shelton, CT, USA). The absorbance readings were made against distilled water as blank in quartz cuvettes of 1-cm path length at room temperature (22 °C). The total monomeric anthocyanin content, expressed as mg cyanidin-3-glucoside/L juice, was calculated using the following equation:

(1) $$\mathrm{TA}=\;\frac{A\cdot \mathrm{MW}\cdot \mathrm{DF}\cdot 1000}{\varepsilon \cdot l}$$

where TA = total anthocyanin content (mg/L) and A = absorbance, calculated as:

A = (A_pH1.0_ − A_pH4.5_)_506 nm_ − (A_pH1.0_ − A_pH4.5_)_700 nm_ (2)

where MW = molecular weight, DF = dilution factor, l = path length, ε = molar extinction coefficient, and 1000 = conversion factor from grams to milligrams.

The radical scavenging capacity of the juice was evaluated by the mean of the free radical 2,2′-azinobis-3-ethyl-benzthiazino-6-sulphonic acid (ABTS) assay [18], which was slightly modified [19]. The freshly prepared free radical ABTS solution was diluted with distilled water to obtain an absorbance of 0.6–0.8 at 734 nm. Subsequently, 6 mL of diluted ABTS solution was added to 0.1 mL of Cornelian cherry juice, and the obtained sample were kept for 15 min at ambient temperature in the absence of light. The capacity of the juice to quench the ABTS^+·^ was spectrophotometrically measured at 734 nm and expressed as µmol Trolox equivalents/L juice by the means of a calibration curve of the Trolox standard.

The total phenolic content of the Cornelian cherries juice was evaluated by the Folin-Ciocalteu assay [20]. Briefly, five-fold diluted juice (0.25 mL) was added to 1.6 mL of Folin-Ciocalteu reagent, and the obtained mixture was neutralized with 1.2 mL of 0.7 M solution of sodium carbonate. The mixture was left to react in the dark at ambient temperature for 2 h and the absorbance was measured at 760 nm. The results were expressed in mg gallic acid equivalents (GAE)/L juice using a calibration curve of the gallic acid standard.

### 2.3. Degradation Studies

To determine the impact of various natural and synthetic sweeteners on the stability of anthocyanins from the Cornelian cherry fruit juice, four formulations of juice were prepared by adding different sweeteners: Sucrose (120 g/L), fructose (80 g/L), aspartame (1 g/L), and acesulfame K (1 g/L). A control sample in the absence of sugars or synthetic noncaloric sweeteners was also used. The thermostability of the pigments in the sweetened juices was also evaluated at 2 °C, 25 °C, and 75 °C. The juice formulations were placed in dark, well-capped glass bottles and kept in the refrigerator (2 °C) or in a water bath at 25 °C and 75 °C equipped with a thermostat. From time to time (0, 2, 4, 8, 10, 22, 24, 26, 28, 31, and 33 h for the juice stored at 75 °C and 0, 20, 24, 48, 144, 168, 192, 216, and 306 h for the samples stored at 2 °C and 25 °C, respectively), juice samples were removed, rapidly cooled in an ice water bath, and the monomeric anthocyanin content was evaluated using the pH differential method. The changes in anthocyanin concentration were fitted into a first-order kinetic model, and the reaction rate constants (k) and the half-lives (t_1/2_) were determined applying the equations given above:

−ln[TA/TA_0_] = kt (3)

t_1/2_ = −ln0.5/k (4)

where [TA] = total anthocyanin content (mg/L) at time t, [TA_0_] = initial total anthocyanin content (mg/L), k = reaction rate constant (h^−1^), t = reaction time (h), and t_1/2_ = half − life (h).

The calculated rate constants were fitted to an Arrhenius type equation in order to determine the temperature effect on the anthocyanin degradation process:

k = K_0_e^−Ea/RT^ (5)

where k = rate constant (h^−1^), K_0_ = frequency factor (h^−1^), E_a_ = activation energy (kJ/mol), R = universal gas constant (8.314 J/mol∙K), and T = absolute temperature (K).

### 2.4. Statistical Analysis

All experiments and measurements were performed in triplicate, and the recorded data is presented as mean values ± standard deviations. Experimental data were subjected to statistical analysis using XLSTAT Release 10 (Addinsoft, Paris, France) software and the significance level was assessed by one-way analysis of variance (ANOVA) with the significance defined at *p* < 0.05.

## 3. Results and Discussion

### 3.1. Fruit Juice Parameters

The underutilized Cornelian cherry fruits present remarkable nutraceutical properties, a fact that enables us to recommend these fruits as valuable source of health-promoting compounds [8,21]. The Cornelian cherry fruit juice could act as functional food and could emerge as a product of special relevance in the nonalcoholic beverages industry due to its high content of bioactive compounds, especially antioxidants [3,8]. The physicochemical characteristics of the obtained Cornelian cherry juice, such as pH and total soluble solids (TSS), were determined for accurate characterization of the juice. The pH value was 3.12 ± 0.04, a low pH characteristic for Cornelian cherry fruits as already reported by other studies [8]. The TSS content determined in the Cornelian cherry juice was 10.03 ± 0.42° Brix, in accordance with previously measured TSS values in Cornelian cherry fruits, which normally range from 15° Brix to 24° Brix for the undiluted juice [7,22]. The free radical scavenging ability of the *Cornus mas* fruit juice was assessed by the ABTS test and was found to be 147.25 ± 7.04 µmol Trolox/L juice.

The phenolic compounds are a major class of bioactive metabolites in the Cornelian cherry fruits. They are demonstrated to possess remarkable in vitro antioxidant activity, conferred mainly by their high hydrogen donating ability and their capacity in chelating metals [23,24]. Therefore, determining the total phenolic content of the fruit juice is an important tool in assessing its antioxidant activity. The TPC of juice obtained from Cornelian cherry fruits was evaluated using a Folin–Ciocalteu assay and was determined to be 2.03 ± 0.86 g GAE/L [20]. Among phenolics, anthocyanins are one of the major constituents of Cornelian cherry fruits, which produce the vivid red color. The total anthocyanin content of the fruit juice was 93.43 ± 4.39 mg Cy-3-glu/L as determined by the pH differential method [25].

**Figure 1:**
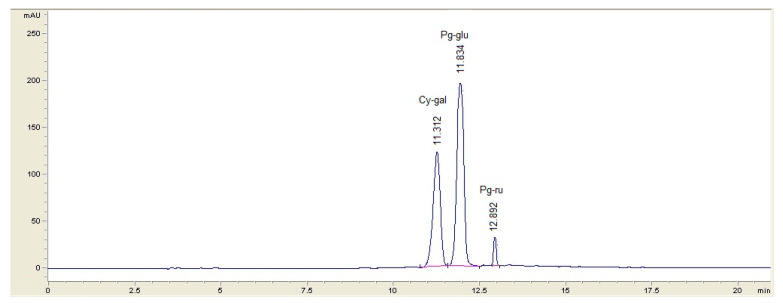
HPLC chromatogram (506 nm) of Cornelian cherry fruit juice.

HPLC analysis coupled with PDA (photodiode array) is the most commonly used analytical tool to identify the anthocyanic profile, which is distinctive for each fruit and cultivar [26]. The quantification of anthocyanins by the pH differential method is a validated, effective, quick, accurate, and simple assay, being extensively used in laboratory determinations as well as in the industry. Lee et al. [26] demonstrated a high correlation between HPLC and the spectrophotometric differential method when quantifying anthocyanins in natural samples. The anthocyanin profile of the investigated juice was studied by HPLC. The analysis demonstrated the presence of cyanidin-3-O-galactoside (Cy-gal), pelargonidin-3-O-glucoside (Pg-glu), and pelargonidin-3-O-rutinoside (Pg-ru) (Figure 1). Our results are in accordance with those obtained by Pawlowska et al. [2], which identified the same three anthocyanin compounds on the basis of HPLC-PDA-MS/MS^n^ analysis. Among the three identified anthocyanins, Pg-glu was the most representative, being found as the main component of the anthocyanin fraction of the juice. Investigating the Cornelian cherry fruits from Anatolia, Turkey, Tural and Koca [6] identified the presence of three different anthocyanins: cyanidin-3-O-glucoside, cyanidin-3-O-rutinoside, and pelargonidin-3-O-glucoside, the last anthocyanin pigment being the predominant anthocyanin as also found in our study. Seeram et al. [27] reported also the presence of three anthocyanin compounds in the fruit of *Cornus mas*: delphinidin-3-O-β-galactopyranozide, cyanidin-3-O-β-galactopyranozide, and pelargonidin-3-O-β-galactopyranozide, while other studies revealed the presence of up to six anthocyanins. Martinovic and Cavoski [7] identified delphinidin-3-O-glucoside, cyanidin-3-O-galactoside, cyanidin-3-O-robinobioside, pelargonidin-3-O-galactoside, and pelargonidin-3-O-robinobioside in some local Montenegro *Cornus mas* cultivars.

The differences in the anthocyanin profile of the *Cornus mas* fruits could be generated by genetic differences between various cultivars/genotypes or growing conditions (geographical origin, soil, and environmental conditions) and also by the ripening degree of the investigated fruits [2].

### 3.2. Degradation Studies

The loss of anthocyanic pigments during juice storage is an important quality factor. The global variation of the anthocyanin concentration in the Cornelian cherry juice was spectrophotometrically assessed along the storage of the juice at three distinct temperatures (2 °C, 22 °C, and 75 °C) when various sweeteners were added. Temperature was the factor that most significantly affected the anthocyanin degradation. During heating, regardless of the added sweeteners, the degradation of the pigments from *Cornus mas* fruits increased by increasing the storage temperature. Kinetic modeling is a valuable tool often used to determine the influence of processing parameters on food quality and safety. An accurate determination of degradation kinetic parameters, such as reaction order, rate constants, half-life, and activation energy, is very important to predict the quality loss by various thermal treatments or storage of food products and is useful to minimize nutrients loss or to enhance the quality of foods. The degradation of anthocyanin was reported to obey first-order kinetic law [28]. The isothermal degradation of the anthocyanins in the investigated juice obeyed a first-order kinetics law, which is in accordance with usually reported anthocyanins’ thermal degradation in red-colored juices [29,30].

**Figure 2:**
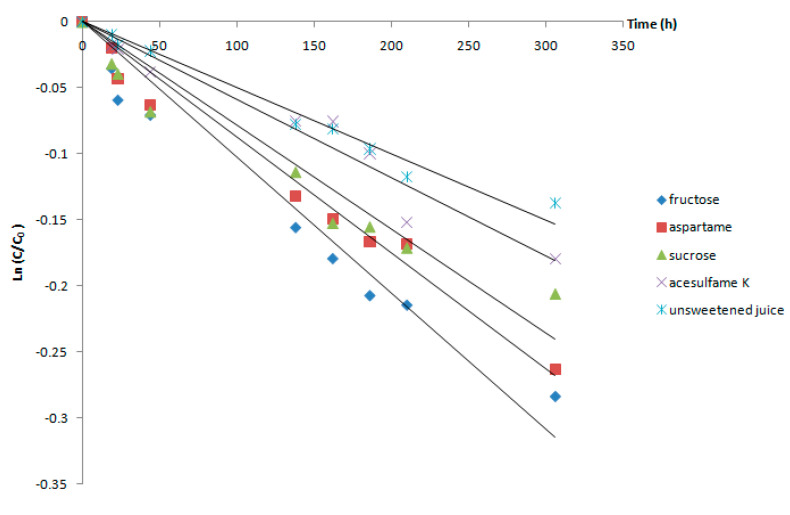
Degradation of the anthocyanins from Cornelian cherry fruit juice at 2 °C.

Anthocyanins from the Cornelian cherry juices stored at low temperature were the most stable. The changes in the total monomeric anthocyanin content of the unsweetened juice stored at 2 °C resulted in the slowest degradation (Figure 2).

After 12 days of storage, the unsweetened Cornelian cherry juice lost 13% of the anthocyanin pigments. The use of various sweeteners accelerated the degradation process. The anthocyanin stability of the juice sweetened with fructose was the lowest, as fructose caused a decrease of 25% in the pigment concentration. Similar results concerning the promoting effect of fructose on anthocyanin degradation have been reported in many other studies [16,31,32].

The kinetic parameters of the anthocyanin degradation process in Cornelian cherry fruit juice during storage are presented in Table 1. The rate constants of the degradation reactions clearly show that this process was affected by the presence of different sweeteners in the matrix. The stability of the pigments in the presence of acesulfame K was 1.75-fold higher than that observed by storage in the presence of fructose. When aspartame was used as a sweetener, the degradation at 2 °C occurred 1.5-fold faster, while the juice storage in the presence of sucrose resulted in an increase of the k value of 1.34-fold, as compared to acesulfame K.

**Figure 3:**
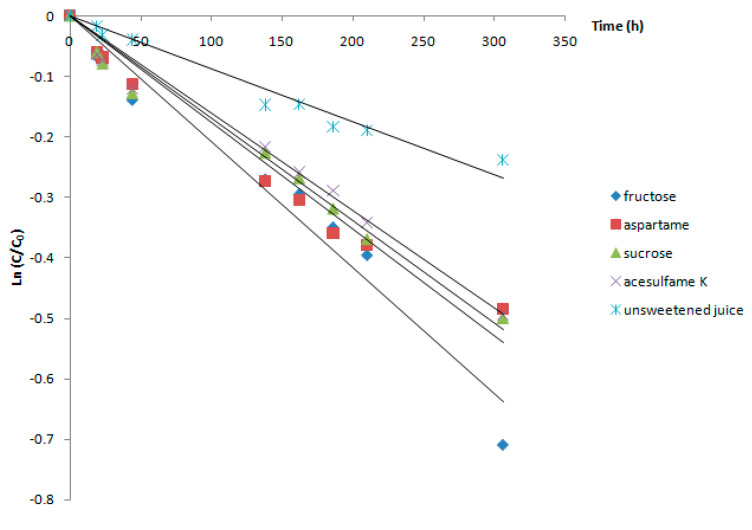
Degradation of the anthocyanins from Cornelian cherry fruit juice at 25 °C.

Increasing the storage temperature to 25 °C (room temperature) caused the stability of the juice pigments to decrease (Figure 3).

After 12 days of juice storage at 25 °C, fructose determined the lowest content and stability of anthocyanic pigments in Cornelian cherry fruit juice. The decrease of anthocyanins stability was also observed in all samples sweetened with sucrose, aspartame, and acesulfame K, compared to unsweetened juice. When acesulfame K was used as sweetener, the stability of the pigments was the highest among all treated samples. The rate constant k, in this case, was not significantly different from that obtained in the case of sucrose and aspartame, but was ~1.2-fold lower than that determined for the degradation of the juice in the presence of fructose.

**Figure 4:**
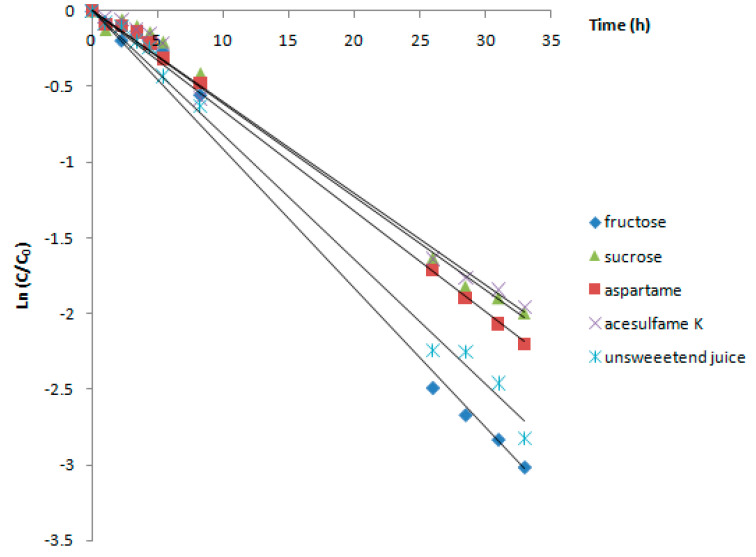
Degradation of the anthocyanins from Cornelian cherry fruit juice at 75 °C.

In order to investigate the effect of heating on the *Cornus mas* anthocyanins’ stability, the juice was subjected to a prolonged exposure at 75 °C, as it is well known that magnitude and period of heating strongly influence the anthocyanin degradation process [28]. Numerous studies reported an accelerated decrease of the anthocyanin content with an increase of temperature [12,33]. In the model experiment performed at 75 °C, the faster degradation was observed. The effect of the sweeteners was different that observed at 2 °C and 25 °C. Increasing the temperature resulted in a protective effect of acesulfame K, aspartame, and sucrose on the anthocyanins from the fruit juice (Figure 4). The degradation in presence of sucrose occurred 1.35-fold slower compared to the unsweetened juice. The only sweetener that maintained the deleterious effect on the anthocyanin stability was fructose, which slightly accelerated the degradation process.

These results are in accordance with those obtained in previous studies, which demonstrated the formation of hydroxymethyl furfural as a reaction product of fructose dehydration, a compound which affects the anthocyanins stability [31,32].

The classic approach used to predict the influence of temperature on degradation processes is the Arrhenius model [34]. The rate constants of anthocyanins’ degradation in the presence of the chosen sweeteners were fitted to an Arrhenius type equation. The obtained activation energies (E_a_) are given in Table 1 and ranged in the interval of 48.72–58.09 kJ/mol. High E_a_ values indicate a high temperature dependence of the pigment degradation reaction [35] and also suggest that a smaller temperature elevation results in a more rapid degradation of the anthocyanins [36]. The highest E_a_ value, and thus the most important influence of temperature on the pigment stability, was determined for the anthocyanins stored in the absence of any sweetener, while using aspartame or sucrose as sweeteners for the juice resulted in the lowest value of the E_a_, suggesting that these two sweeteners determined a lower susceptibility to anthocyanins degradation at high temperatures. The obtained E_a_ values for the Cornelian cherry anthocyanins thermal degradation are in accordance to those reported in previous studies, which were found to be in the range of 55.96–58.55 kJ/mol [37,38].

## 4. Conclusions

The present study investigated the thermal stability of Cornelian cherry fruit anthocyanins in model juice systems in the presence of four commonly used food sweeteners. Sucrose, fructose, aspartame, and acesulfame K significantly affected the anthocyanin degradation process in the juice. Fructose presented the highest promoting effect on the pigment degradation process at all the investigated temperatures (2 °C, 25 °C and 75 °C), accelerating this reaction ~2 times at 2 °C and 25 °C. At high temperatures, as also suggested by the obtained E_a_ values, using sucrose as a sweetener had a positive effect on the stability of anthocyanins from Cornelian cherry juice.

## Figures and Tables

**Table 1:** Kinetic parameters of Cornelian cherry anthocyanins degradation in sweetened and unsweetened juice at different temperatures.

Sample	Temp. (°C)	k·10^−3^ (h^−1^) ^1^	t_1/2_ (h) ^2^	E_a_ (kJ/mol)
Unsweetened juice	2	0.50 (0.9746)	1386 ^a^	58.09
	25	0.87 (0.9647)	796.6 ^d^	
	75	82.17 (0.9944)	8.4 ^g^	
Juice + sucrose	2	0.79 (0.9080)	877.2 ^b^	48.72
	25	1.69 (0.9726)	410.1 ^e^	
	75	61.30 (0.9905)	11.3 ^h^	
Juice + fructose	2	1.03 (0.9499)	672.8 ^c^	50.69
	25	2.08 (0.9643)	333.2 ^f^	
	75	91.65 (0.9890)	7.6 ^g^	
Juice + aspartame	2	0.88 (0.9732)	787.5 ^b^	48.75
	25	1.77 (0.9644)	391.5 ^e^	
	75	66.12 (0.9972)	10.5 ^h^	
Juice + acesulfame K	2	0.59 (0.9490)	1174.6 ^a^	51.75
	25	1.61 (0.9740)	430.4 ^e^	
	75	60.35 (0.9918)	11.5 ^h^	

^1^ The determination coefficients (*R*^2^) are given in parentheses; ^2^ Different superscript letters are used to designate values which are statistically significant (*p* < 0.05).
